# A new encryption algorithm for image data based on two-way chaotic maps and iterative cellular automata

**DOI:** 10.1038/s41598-024-64741-x

**Published:** 2024-07-19

**Authors:** Mimouna Abdullah Alkhonaini, Entesar Gemeay, Firas Muhammad Zeki Mahmood, Mohamed Ayari, Farhan A. Alenizi, Sangkeum Lee

**Affiliations:** 1 Department of Computer Science, College of Computer and Information Sciences, Prince Sultan University, Riyadh, Saudi Arabia; 2 Department of Electronics and Communication Engineering, College of Engineering, Tanta University, Tanta, Egypt; 3 Department of Computer Engineering, Computer and Information Technology College, Taif University, Taif, Saudi Arabia; 4 Department of Communication and Computer Engineering, Cihan University-Erbil, Erbil, Kurdistan Region Iraq; 5 Department of Information Technology, Faculty of Computing and Information Technology, Northern Border University, Arar, Saudi Arabia; 6 Syscom Laboratory, National Engineering School of Tunis, University of Tunis El-Manar, Tunis, Tunisia; 7 Electrical Engineering Department, College of Engineering, Prince Sattam Bin Abdulaziz University, 11942 Al-Kharj, Saudi Arabia; 8 Computer Engineering, Hanbat National University, Daejeon, 34158 South Korea

**Keywords:** Image encryption, Cryptography, Reversible cellular automata, Spatiotemporal chaos, Computational science, Computer science, Information technology

## Abstract

Due to their simplicity of implementation and compliance with the encryption issue, chaotic models are often utilized in picture encryption applications. Despite having many benefits, this approach still has a crucial space issue that makes encryption algorithms based on it susceptible to brute-force assaults. This research’s proposed novel picture encryption technique has a vast key space and great key sensitivity. To achieve this goal, the proposed method combines two-way chaotic maps and reversible cellular automata (RCA). First, this approach uses a two-way chaotic model named spatiotemporal chaos for image confusion. This step includes permuting the image pixels using a chaotic map at the byte level. Then, the RCA model is utilized for image diffusion. In this step, the RCA model iterates over image pixels to modify them at the bit level. The method’s performance in encrypting grayscale images was evaluated using various analysis methods. According to the results, the proposed method is a compelling image encryption algorithm with high robustness against brute-force, statistical, and differential attacks.

## Introduction

Today, a considerable amount of information is constantly being exchanged through different media and networks. In the meantime, images can be considered one of the most widely used types of media being exchanged due to their structure and the human perceptual system; it can also be considered the fastest medium for conveying concepts^1^. Images being exchanged in computer networks can contain information about security, finances, etc. In such a situation, the need to provide solutions for improving multimedia information security is felt more than ever. Ensuring the security of multimedia data is one of the goals of forming a branch of security called “multimedia security”^2^. Researchers of multimedia security are always looking for more secure methods to encrypt multimedia data. An efficient algorithm for image encryption can be helpful in various applications such as video conferences, image databases, cloud storage spaces, military applications, etc.^3^.

Image encryption is a method to secure information transmission despite security threats such that only authenticated people can interpret it. A cryptographic method requires solving many problems, such as data transformation, authentication, and key distribution^4^. With the development of internet-based environments, information security is becoming more critical every moment. Generally, data encryption can protect users’ information during transmission in public channels. However, conventional methods for data encryption have limitations when used in images. Some of these issues include low efficiency in working with big data, high correlation between image pixels, etc.^5^. Chaos theory^6^, by presenting a complex nonlinear system, has suitable characteristics for encryption applications, including image encryption, and can provide some requirements for efficient image encryption. However, one of the shortcomings of the encryption techniques developed by this idea is the crucial space constraint, which leaves them open to brute-force assaults^7^.

This work overcomes these drawbacks by putting forth a novel method of image encryption that makes use of the advantages of reversible cellular automata (RCA) and chaotic maps. We have two things to contribute:

1. *Enhanced chaotic model* To increase the chaotic model’s effectiveness in picture encryption applications, we present a brand-new model that is based on spatiotemporal chaos theory. The goal of this model is to solve the drawbacks of traditional chaotic models for image encryption.
2. *Diffusion technique with RCA* To tackle the enduring correlation problem between encrypted image pixels, we incorporate a diffusion technique based on RCA. This work is a significant advance as it combines RCA diffusion with an improved chaotic model, a combination not previously explored in research.

The suggested method combines these strategies to produce higher resistance to the correlation between picture pixels, a wider key space, and increased robustness against multiple attacks.

Section two studies relevant works, and section three describes the suggested approach for picture encryption. The implementation and evaluation findings are reported in the fourth part, and conclusions are drawn in the fifth section. Finally, several recommendations for more study in this area have been made.

## Related works

A Tent-Dynamics Coupled Map Lattices (TDCML) and Household Diffusion-based picture encryption technique is proposed in Ref.^8^. Confusion and diffusion are the first two phases of this method. The picture is first permuted using a cyclic change method, and then the chaotic sequence is generated using the TDCML system. After that, the Household orthogonal decomposition technique is used for image diffusion. Reference^9^ presents a quick picture encryption technique based on the lifting scheme and chaotic model. The components of these two sets are then progressively confused using pseudo-random sequences based on the chaos model. The picture is then encrypted using the lifting algorithm. Reference^10^ proposes a hidden attractor chaotic system-based and Knuth–Durstenfeld algorithm-based picture encrypting scheme. Additionally, the Knuth–Durstenfeld approach may improve the complexity of the permutation space while reducing time complexity in this system and exhibiting adequate pseudo-random behavior^11^. The TMDPCML system proposed in this research improves the spatial–temporal correlation of the chaotic system and effectively increases the diffusion algorithm’s efficiency. In addition, the TMDPCML system has a wider key space and more chaotic behavior. Reference^12^ presents a dual image encryption technique based on chaos theory and convolutional neural networks (CNN). The CNN convolution is then used in a chaotic sequence to create the picture confusion indicator. Reference^13^ presents a technique for encrypting grayscale and color medical photos. This study introduces a novel method of picture segmentation based on block structure. In Ref.^14^, a DNA coding and Sine-Piecewise Linear Chaotic Map (SPWLCM)-based picture encryption technique is proposed. In this research, SPWLCM is used to improve the performance of the traditional chaos map in the confusion step.

In Ref.^15^, an encryption algorithm based on chaos and truth table is presented. This algorithm uses nonlinear chaos sequences for confusion in horizontal, vertical, and diagonal directions; and for diffusion in two directions. In the diffusion step, two matrices are used to change the values of pixels: one is the matrix processed by the scrambler, and the other is the matrix produced by the truth table. In Ref.^16^, the two-dimensional chaotic map is used for image encryption. The chaos model proposed in this research tries to improve the limitations of conventional chaotic systems and presents a two-dimensional chaotic map through two one-dimensional chaos maps in the form of a linear function.

Reference^17^ proposes a medical picture encryption technique based on a three-leaf, five-dimensional chaotic system and genetic operation. This algorithm adheres to the confusion-diffusion paradigm, much like most chaos-based encryption methods. The chaotic matrix is created using this approach, incorporating the five-dimensional three-leaf chaos system and the DNA recombination concept. Also, DNA mutation operation is used at the bit level for the diffusion step. In Ref.^18^, a multi-image encryption algorithm based on Haar wavelet transform and 3D shuffling scrambling is proposed. This research also suggests a three-dimensional confusion algorithm in which the permutation cube is divided and reorganized.

Image Steganography offers an alternate method of concealing information. The security issues with conventional steganography techniques—where incorporating secret data can change the distribution of the cover image—were discussed by researchers in Ref.^19^. They provide a brand-new method that makes use of both colorization and de-colorization. By embedding confidential information during the color conversion process, this method offers a more secure way to conceal data within cover images while also effectively counteracting the typical embedding effect.

Research on Chaotic Image Encryption is still very much in demand. A new technique using a Spiral-Transform-Based Fractal Sorting Matrix (STFSM) was presented by researchers in Ref.^20^. The erratic and repetitive characteristics of STFSM provide superior picture scrambling during encryption. The study includes security assessments showing robust encryption capabilities, as well as a description of the theory and application of STFSM. Nonetheless, more research on particular assault resistance is necessary.

The Double Parameters Fractal Sorting Matrix (DPFSM) was proposed by researchers in Ref.^21^. Because of its distinct self-similar structures and enhanced periodic law, DPFSM is superior to regular matrices and is therefore more appropriate for information security applications. The efficacy of the picture encryption technique based on DPFSM is presented by the authors. Even if the results point to a possible application value, the evaluation would be strengthened by comparison with other matrix-based fractal sorting techniques. Table 1 summarizes the studied works.

**Table 1:** Summary of the literature.

Ref.	Year	Research goal	Method	Limitation
^8^	2020	Image encryption	Tent-dynamics coupled map lattices (TDCML) and household diffusion	Limited key space, potential vulnerability to brute-force attacks
^9^	2020	Fast image encryption	Lifting scheme and chaotic model	May not achieve strong confusion and diffusion properties
^10^	2020	Image encryption	Hidden attractor chaotic system and Knuth-Durstenfeld algorithm	Potential limitations in diffusion effectiveness
^11^	2021	Image encryption	Piecewise Coupled Map Lattice with multi dynamic coupling coefficient	Complexity of implementation compared to simpler chaotic maps
^12^	2021	Double image encryption	Chaos theory and convolutional neural networks (CNN)	Computational cost associated with CNNs
^13^	2021	Medical image encryption	Novel block-based image segmentation and chaotic system	Limited details on specific attacks addressed
^14^	2021	Image encryption	DNA coding and Sine-Piecewise Linear Chaotic Map (SPWLCM)	Potential trade-off between security and encryption speed
^15^	2021	Image encryption	Chaos and truth table	Lacks detailed analysis of diffusion effectiveness
^16^	2021	Image encryption	Two-dimensional chaotic map	May not achieve sufficient key space compared to more complex chaotic systems
^17^	2021	Medical image encryption	Three-leaf, five-dimensional chaotic system and genetic operation	High computational complexity for medical image applications
^18^	2022	Multi-image encryption	Haar wavelet transform and 3D shuffling scrambling	Potential limitations in resisting chosen-plaintext attacks
^19^	2023	Image steganography	De-colorization and colorization for embedding secret information	Focuses on steganography, may not directly address encryption challenges
^20^	2022	Chaotic image encryption	Spiral-transform-based fractal sorting matrix (STFSM)	Requires further investigation into resistance against specific attacks
^21^	2021	Image encryption	Double parameters fractal sorting matrix (DPFSM)	Lacks comparison with other fractal sorting matrix-based encryption methods

## Proposed method

The explanation of the suggested encryption algorithm is the focus of this section. The recommended technique encrypts images using a fresh spatiotemporal chaotic model. Therefore, the proposed chaos model will be described first, and then the steps of image encryption using this model will be presented.

### Proposed spatiotemporal chaos model

Spatiotemporal chaos is substantially more complicated in behavior and exhibits more pseudo-random properties than linear chaos systems. In the proposed algorithm using CML and based on the model proposed in Ref.^22^, a method of permuting image pixels based on spatiotemporal chaos theory is presented. In the following, we will describe the proposed chaos model.

The nonlinear chaotic algorithm (NCA) is generated based on the logistic map. A logistic map can be defined based on the following equation:

1 $${x}_{n+1}=\mu {x}_{n}\left(1-{x}_{n}\right), n=\mathrm{1,2},3,\dots .$$

**Figure 1:**
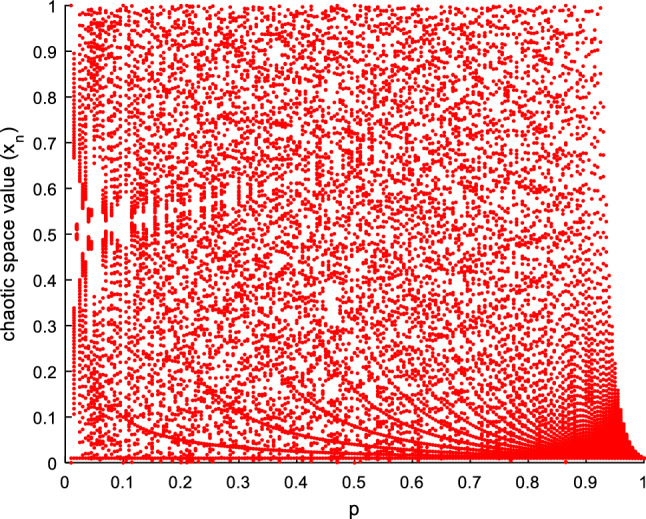
Chaotic map resulting from Eq. (2) based on p key values.

In the above equation, if $3.57\le \mu \le 4$, then the logistic map will show a chaotic behavior. One of the disadvantages of using this model is its small critical space and, as a result, its low security. Therefore, in several research studies, efforts have been made to overcome these disadvantages by providing more efficient models. Including:

2 $$ x_{{n + 1}}  = \left\{ {\begin{array}{*{20}l}    {\frac{{x_{n} }}{p}} \hfill & {0 \le x_{n}  \le p} \hfill  \\    {\frac{{1 - x_{n} }}{{1 - p}}} \hfill & {p < x_{n}  \le 1} \hfill  \\   \end{array} } \right.,\;n = {\mathrm{1,2}},3, \ldots , $$

where ${x}_{n}\epsilon (\mathrm{0,1})$ represents the *n*th term of the chaotic sequence, and *p* specifies the key. In the above equation, if $p\in (\mathrm{0,1}]$, the sequence *x* will have a chaotic behavior. In Fig. 1, the structure of the chaotic map resulting from Eq. (2), for various values of key *p* is displayed.

Figure 1 displays the chaos map for the primary key x_0_ = 0.01. Based on this figure, the chaotic map obtained from Eq. (2) also faces several problems. The key space in this sequence is also within the interval [0, 1], and patterns of functional behavior can be seen in this chaotic map (for p > 0.85). On the other hand, if the key *p* and the initial term ${x}_{0}$ are equal, the chaos sequence will be a unit vector.

CML serves as a model for dynamic systems featuring discrete space and position, exhibiting successive states. It is commonly utilized as a foundational tool for exploring dynamics within spatiotemporal chaotic systems.

A two-way CML system can be modeled as the following equation^22^:

3 $$\left\{\begin{array}{l}{x}_{n+1}=\left(1-\varepsilon \right)f\left({x}_{n}\left(i\right)\right)+\frac{\varepsilon }{2}\{f({x}_{n}\left(i-1\right))+f({x}_{n}(i+1))\} \\ f(x)=\mu x\left(1-x\right) \end{array}\right..$$

**Figure 2:**
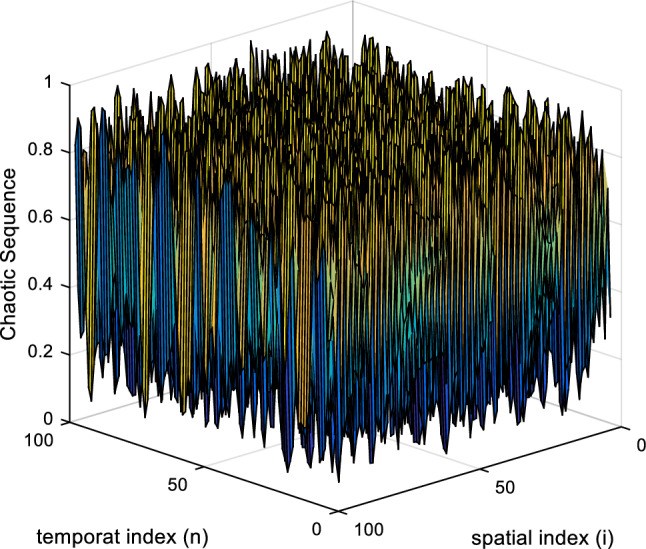
The attractor of the proposed NCA-based CML.

Also, $3.57\le \mu \le 4, 0<x<1 , 0<f\left(x\right)<1$ defines the range of values that can be used in the Eq. (3). Equation (2) can be replaced in Eq. (3) to take advantage of more complex systems. By performing this action, a spatiotemporal chaos model can be defined as the following equation:

4 $${x}_{n+1}=\left(1-\varepsilon \right)f\left({x}_{n}\left(i\right)\right)+\frac{\varepsilon }{2}\left\{f\left({x}_{n}\left(i-1\right)\right)+f\left({x}_{n}\left(i+1\right)\right)\right\},$$

$$f\left( x \right) = \left\{ {\begin{array}{*{20}l}    {\frac{x}{p}} \hfill & {0 \le x \le p} \hfill  \\    {\frac{{1 - x}}{{1 - p}}} \hfill & {p < x \le 1} \hfill  \\   \end{array} } \right.,$$

where f(x) represents the nonlinear chaotic function in Eq. (2). The attractor of the proposed NCA-based CML to encrypt images is shown in Fig. 2.

The proposed spatiotemporal chaotic system, by considering two key values *ε* and *p* as well as expanding the chaotic system to time and space, can provide a much higher level of security for information encryption. Because it will be much more difficult for attackers to understand the behavioral model of the system in spatiotemporal chaotic systems.

### The proposed encryption algorithm based on spatiotemporal chaos and RCA

Confusion and diffusion are the two key stages of the suggested method. The created permutation sequence in the confusion phase moves the pixels of the picture using the suggested spatiotemporal chaotic system. Then, in the diffusion phase, an RCA is applied iteratively on the more valuable half of the bits of each image pixel to change its value. After these two steps, the final encrypted image will be obtained.

We’ll go through the suggested algorithm’s encryption processes in the following paragraphs. We assume that the intended picture for encryption has dimensions of 256 $\times$ 256 pixels to avoid decreasing the generality of applying the suggested approach. The following steps describe the necessary steps for encrypting the assumed image using the proposed method:

- *Step 1* The input image matrix of ${X}_{256\times 256}$ is converted into a one-dimensional vector as $M=\{{m}_{1},{m}_{2},\dots ,{m}_{256\times 256}\}$.
- *Step 2* Using the following equation, the initial diffusion is performed:
  5 $$\left\{\begin{array}{l}{m}_{1}^{\prime}={m}_{256\times 256}\oplus {m}_{1 } \\ {m}_{j+1}^{\prime}={m}_{256\times 256-j}\oplus {m}_{j}^{\prime}, j=\mathrm{1,2},\dots ,256\times 255\end{array} ,\right.$$
  where the ⊕ operator indicates the bitwise XOR operation. The result of this operation will be a vector as $M^{\prime}=\left\{{m^{\prime}}_{1},{m^{\prime}}_{2},\dots ,{m^{\prime}}_{256\times 256}\right\}$.
- *Step 3* In the third step, calculate the sum of the values in the matrix $M^{\prime}$ and by successively dividing that number by 10, we transfer it to the range of [0, 1]. For example, we convert the sum of 2,564,453 to 0.2564453. Then, use the obtained number as the initial value of x_1_ in Eq. (2). The start of sequence is chosen from a specific location in the chaotic sequence (such as the hundredth element: N_s_ = 100) and the desired length is extracted from the sequence (${N}_{s}+256\times 256$). We will consider this sequence as $A=\{{a}_{1},{a}_{2},\dots ,{a}_{256\times 256}\}$.
- *Step 4* Sort the sequence *A* in ascending order to produce the permutation sequence *IX*. The permutation sequence *IX* specifies the order in which each of the elements of *A* is arranged, and in other words, it defines the order in which the elements of *A* are placed in the new sorted sequence. By applying the *IX* permutation sequence on the sequence *M’* (step 2), the confused sequence ${M}^{{\prime\prime}}$ is obtained as ${M}^{{\prime \prime}}=\left\{{m^{\prime}}_{IX(1)},{m^{\prime}}_{IX(2)},\dots ,{m^{\prime}}_{IX(256\times 256)}\right\}$. Then the vector M’’ is converted to the matrix form as $MM=\left\{m{m}_{i,n}\right|i,n=\mathrm{1,2},\dots ,256\}$.
- *Step 5* Using Eq. (4), the spatiotemporal chaotic matrix $X=\left\{{x}_{n}\left(i\right)|i,n=\mathrm{1,2},\dots ,400\right\}$ is generated, (which is shown in Fig. 2) and Then we convert it to $Y=\left\{y{}_{i.n}|i,n=\mathrm{1,2},\dots ,400\right\}$ using the following equation:
  6 $$y_{i.n} = \left\lfloor {(x_{n} \left( i \right) \times 10^{17} ) mod256} \right\rfloor .$$
  It is obvious that: $y{}_{i.n}\in \left[\mathrm{0,256}\right]$. Also, using the following equation, sequence *X* is converted into sequence *Z*:
  7 $$z_{i.n} = \left\lfloor {(x_{n} \left( i \right) \times 10^{17} ) mod2} \right\rfloor .$$
  Since $z{}_{i.n}\in \{\mathrm{0,1}\}$, this binary vector is used as the initial input of the RCA.
- *Step 6* Using the following equation, the second diffusion is applied on the $MM$ matrix:
  8 $${c}_{i,n}=m{m}_{i,n} \oplus y{}_{{N}_{s}+i.{N}_{s}+n}.$$

In the next step, RCA will be used to modify the bits of each pixel in the image. The RCA model introduced in Ref.^23^ is used in this step. To save memory and execution time, we consider only the four most valuable bits in each pixel as modifiable data because more than 95% of the information of each pixel is stored in the four most valuable bits of that pixel.

A cell is created in the automata for each pixel in the image so that a two-dimensional RCA with dimensions of $256\times 256$ is produced. Each cell of RCA first converts the value in its corresponding pixel to binary and then stores the four most valuable bits. Since in RCA, the previous state of each cell must always be available, for the initial states of the cell, we use the $z{}_{i.n}$ matrix in Eq. (7) as follows:

9 $${C}_{i,j}^{t0}={z}_{{N}_{s}+i,{N}_{s}+j}.$$

The local rules for determining the next state of each RCA cell are shown in Table 2. In this table, the meaning of each bit is as follows:

- ${S}_{i,j-1}^{t}$: the current bit in the left neighbor of the current cell.
- ${S}_{i-1,j}^{t}$: the current bit in the upper neighbor of the current cell.
- ${S}_{i,j}^{t}$: the current bit in the current cell.
- ${S}_{i+1,j}^{t}$: the current bit in the lower neighbor of the current cell.
- ${S}_{i,j+1}^{t}$: the current bit in the right neighbor of the current cell.
- ${S}_{i,j}^{t-1}$: previous state of the cell for the current bit.
- ${S}_{i,j}^{t+1}$: Next state of the cell for the current bit.

**Table 2:** RCA rules for determining the next state of each cell.

${S}_{i,j-1}^{t}{S}_{i-1,j}^{t}{S}_{i,j}^{t}{S}_{i+1,j}^{t}{S}_{i,j+1}^{t}$	${S}_{i,j}^{t+1}$		${S}_{i,j-1}^{t}{S}_{i-1,j}^{t}{S}_{i,j}^{t}{S}_{i+1,j}^{t}{S}_{i,j+1}^{t}$	${S}_{i,j}^{t+1}$	
	${S}_{i,j}^{t-1}=0$	${S}_{i,j}^{t-1}=1$		${S}_{i,j}^{t-1}=0$	${S}_{i,j}^{t-1}=1$
00000	1	0	10000	0	1
00001	1	0	10001	0	1
00010	1	0	10010	0	1
00011	1	0	10011	0	1
00100	0	1	10100	0	1
00101	0	1	10101	0	1
00110	1	0	10110	1	0
00111	1	0	10111	1	0
01000	0	1	11000	0	1
01001	0	1	11001	0	1
01010	0	1	11010	1	0
01011	0	1	11011	1	0
01100	1	0	11100	0	1
01101	1	0	11101	0	1
01110	1	0	11110	0	1
01111	1	0	11111	0	1

**Figure 3:**
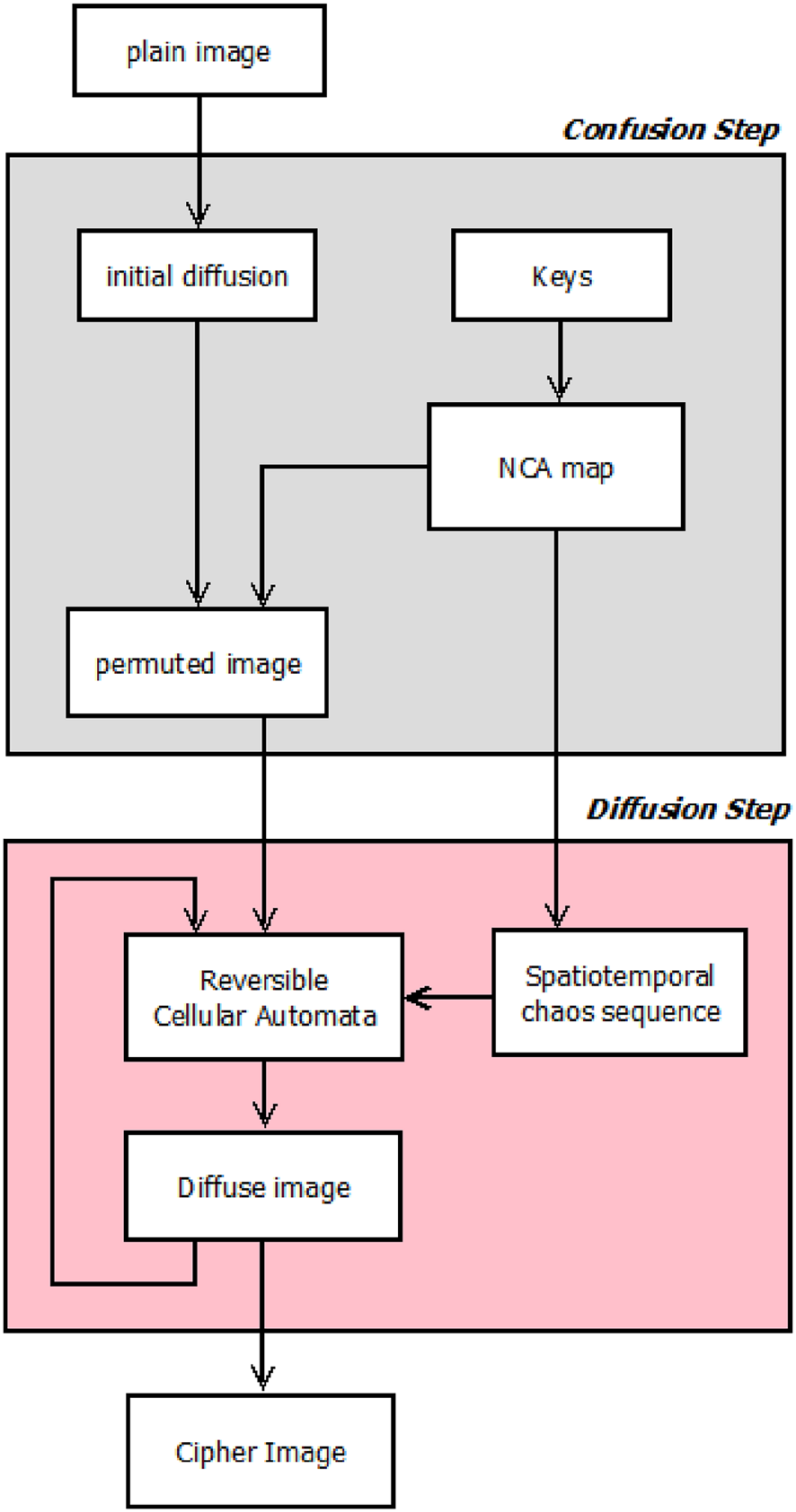
Block diagram of the proposed encryption algorithm.

Von Neumann’s model has been used as a neighborhood determination model in RCA. Each automata cell performs RCA state modification for each of its four bits and through Table 2 rules. This action is repeated *r* times. After repeating *r* times, the four modified bits in each cell (${C}^{{t}_{r}}$) are combined with the four least valuable bits of the image (${c}_{i,n}$) (see Fig. 3).

**Figure 4:**
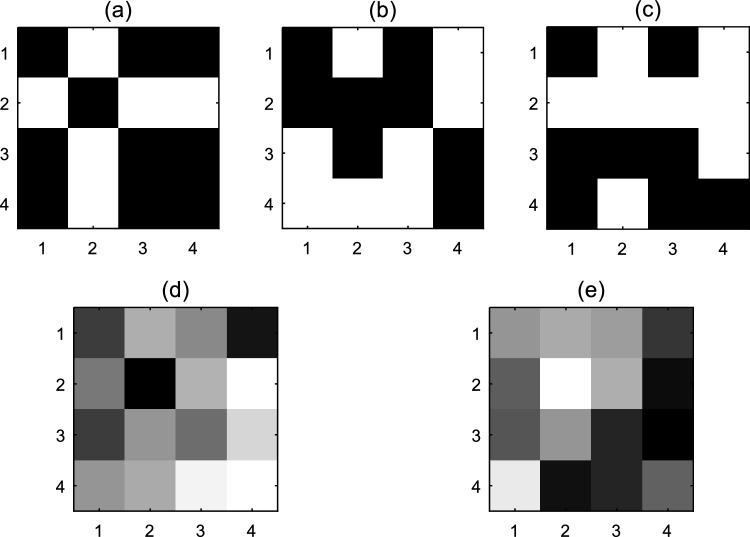
Encryption steps of a 4 × 4 image.


In Fig. 4a, the initial image, which is a 4 × 4 matrix, is displayed. After converting the matrix of the image into a vector and performing initial diffusion using Eq. (5), the image (Fig. 4b) is obtained. To better display the changes, each output is shown as a matrix. For this figure, the total value calculated equals the number 6. By successively dividing this number by 10, the value is 0.6. We use this value as the initial value of x_1_ in Eq. (2) to generate the chaotic sequence in step 3. Image (Fig. 4c) is obtained after sorting the chaotic sequence and permuting the image pixels based on the sorting pattern in step 4. In the next step, using Eq. (4), the spatiotemporal chaotic sequence is generated, and then the sequences $y{}_{i.n}$ and $z{}_{i.n}$ are calculated by Eqs. (6) and (7). The diffusion process is performed using Eq. (8), and its result is illustrated in the image (Fig. 4d). The last step is to use RCA to create the encrypted image. For convenience, we examine the changes made using RCA in the pixel located in the second row and column of matrix (d). The matrix in image (d) contains the following values (see Fig. 4a–e).

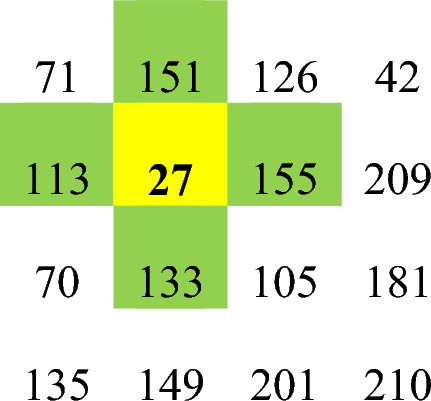


**Figure 5:**
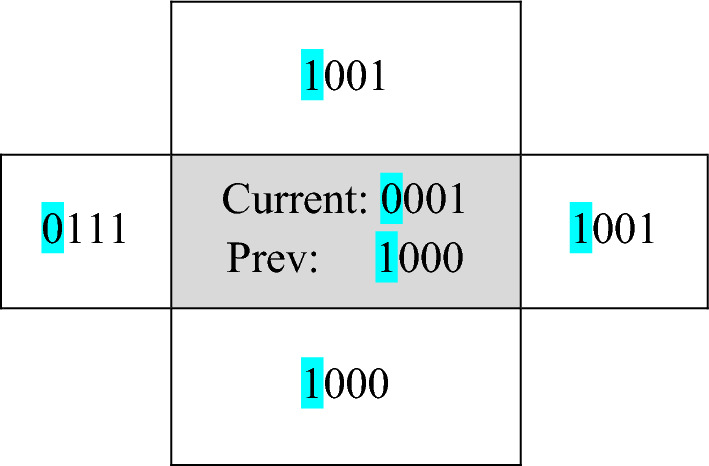
An example of pixel value modification by RCA.

The mentioned pixel has a value of 27. The binary value of this pixel is 00011011. As mentioned, we consider the four most valuable bits in the proposed method. Also, the previous value of the cell in the first iteration is extracted using the sequence ${z}_{i,n}$ and it will have the value of 1100. Now, using Table 2, we determine the next state of each cell. For each bit in the cell, use the rules in Table 2 to obtain its next states. In the mentioned example, we check the most valuable bit in the desired cell. Figure 5 shows the state of the cell in the second row and column as well as the neighbors of the cell in RCA.

In Fig. 5, we consider the state change rules for the most significant bit of the cell shown in the figure. In this cell, the most significant bit is 0. The left neighbor in the corresponding bit has the value of 0, the right neighbor has the value of 1, the upper neighbor has the value of 1, and the lower neighbor has the value of 1. Therefore, the resulting sequence to determine the next state of the desired bit is as follows:

$${S}_{i,j-1}^{t}{S}_{i-1,j}^{t}{S}_{i,j}^{t}{S}_{i+1,j}^{t}{S}_{i,j+1}^{t}= 0 1 0 1 1,$$

$${S}_{i,j}^{t-1}=1.$$

Therefore, based on Table 2, we will have:

$${S}_{i,j}^{t+1}=1.$$

**Figure 6:**
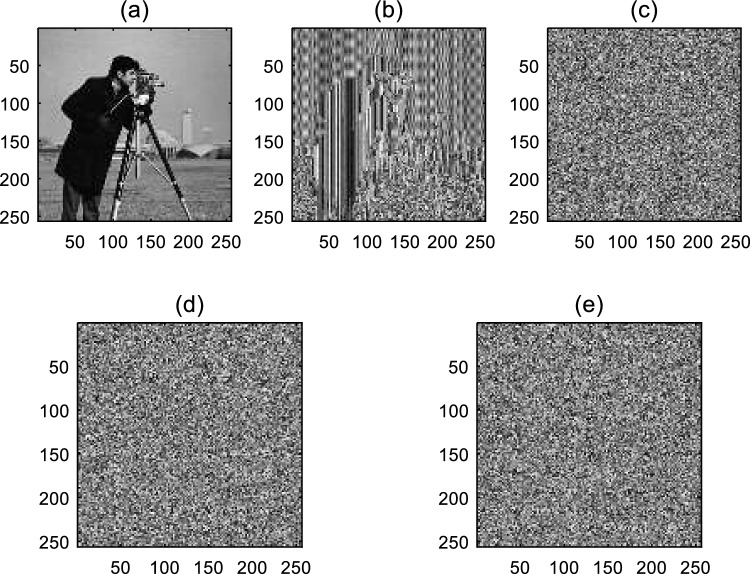
Encrypting the cameraman image using the proposed method.

The values of other bits in each cell are determined similarly. The steps of encrypting the cameraman image are shown in Fig. 6.

## Implementation and results

**Figure 7:**
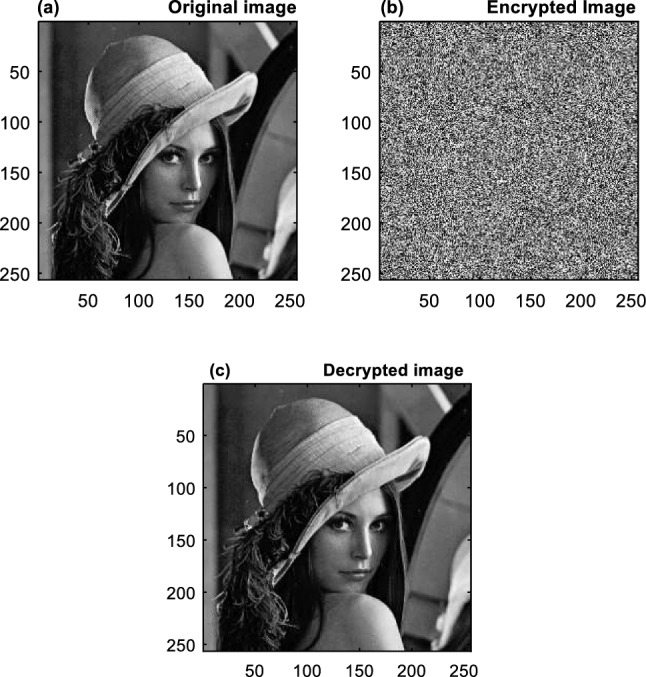
Implementation results: (**a**) input image, (**b**) encrypted image, (**c**) decrypted image.

The proposed encryption algorithm was implemented using MATLAB 2019a software. The proposed method was evaluated using a dataset including 25 grayscale images with size of $256\times 256$. An example of these images is shown in Fig. 7a. Encryption is carried out using the suggested technique in this image. In Fig. 7c, the picture produced by the decryption procedure is likewise shown. Applying the proposed encryption algorithm on all images showed that the proposed algorithm can perform the decryption process without any reduction in quality or changes in the characteristics of the original image.

This feature will not affect the generality of the proposed method, and by applying the proposed algorithm to each layer in color images separately, it can be used to encrypt images with any color system.

### Histogram analysis

**Figure 8:**
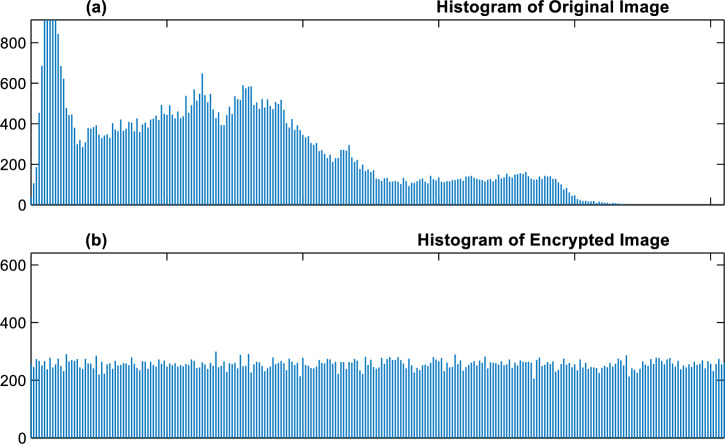
Histogram analysis: (**a**) Lena image initial histogram, (**b**) histogram of encrypted image.

Analyzing the histogram of encrypted photographs allows an attacker to gain crucial information needed to retrieve the original image. These assaults are called statistical attacks. The histogram of the Lena picture is shown in Fig. 8a.

In general, the average standard deviation of the histograms obtained by the proposed encryption algorithm for all test images equals 13.462. Meanwhile, the same criterion for methods^15–17^ is equal to 28.540, 19.582, and 21.867, respectively. Therefore, the proposed algorithm can provide better security against statistical attacks, and useful information about the initial image cannot be obtained by analyzing the histogram of the encrypted image (see Fig. 8b).

### Correlation analysis

Each image pixel in a typical image often has a strong correlation with its nearby pixels, which an attacker may take advantage of. In this experiment, we choose 1000 nearby pairs of pixels from the original picture and the encrypted image (vertical, horizontal, or diagonal neighbors), and we then compute the correlation coefficients using the formula shown in equation^24^:

10 $${r}_{xy}=\frac{cov(x,y)}{\sqrt{D\left(x\right).D(y)}},$$

where, cov(x,y) is calculated as follows^24^:

11 $$cov\left(x,y\right)=\frac{1}{N} \sum_{i=1}^{N}({x}_{i}-\overline{x })({y}_{i}-\overline{y }),$$

12 $$D(x)=\frac{1}{N} \sum_{i=1}^{N}{\left({x}_{i}-\overline{x }\right)}^{2}.$$

**Figure 9:**
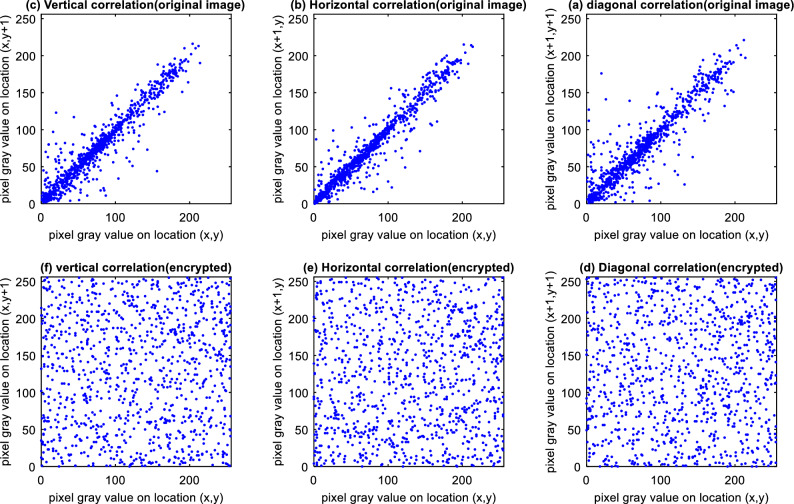
Analysis of the Lena image’s neighboring pixels’ correlation: (**a**) diagonal correlation between neighbor pixels of the encrypted image (**e**) horizontal correlation between neighbor pixels of the encrypted image (**f**) vertical correlation between neighbor pixels of the encrypted image (**b**) correlation between neighbor pixels of the original image on the horizontal axis (**c**) correlation between neighbor pixels of the original image vertically.

The correlation distribution of the Lena image (Fig. 7) before and after encryption has been calculated for various neighborhood states (horizontal, vertical, and diagonal neighborhood). The results of this evaluation are shown in Fig. 9.

Encrypting the image shows that the strong correlation between neighboring pixels is significantly reduced by the proposed encryption algorithm (Table 3). In this Table, the values obtained for the proposed method’s correlation coefficient are compared with those obtained from encryption algorithms proposed in Refs.^15–17^. This means that the probability of extracting meaningful connections between data values encrypted by the proposed method is much lower than compared methods (see Table 3).

**Table 3:** Correlation between neighbor pixels before and after image encryption.

	Proposed	Ref^15^	Ref^16^	Ref^17^
Lena				
Horizontal	− 0.0290	0.0637	0.0214	0.0414
Vertical	0.0080	0.0166	0.1090	0.0192
Diagonal	− 0.0421	− 0.0527	0.0050	0.0533
Camera man				
Horizontal	− 0.0107	− 0.0076	− 0.0560	0.0145
Vertical	− 0.0007	0.0588	0.0007	0.0015
Diagonal	0.0077	− 0.0355	− 0.0574	0.0174
Peppers				
Horizontal	0.0207	0.0204	− 0.0252	0.0218
Vertical	0.0028	0.0553	0.0139	0.0087
Diagonal	− 0.0103	0.0140	0.0023	0.0199
Dataset average (abstract)				
Horizontal	0.0175	0.0306	0.0342	0.0259
Vertical	0.0091	0.0436	0.0412	0.0098
Diagonal	0.0137	0.0341	0.0216	0.0302

### Differential analysis

**Figure 10:**
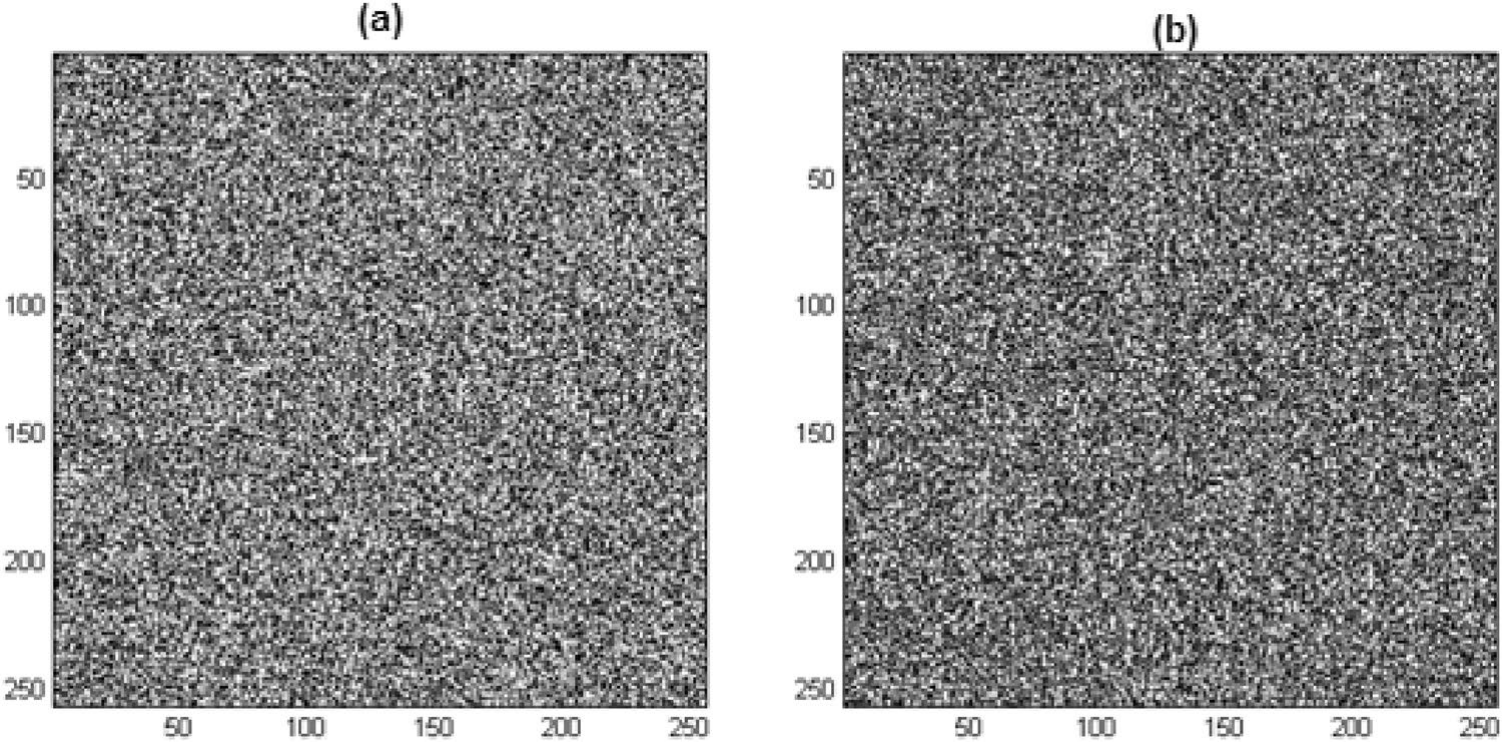
Differential analysis: (**a**) encrypted Lena image, (**b**) encryption result by changing one bit in the original image.

In this experiment, the fourth row and seventh column of Lena’s image’s pixel, which contains the most crucial bit, is the sole bit that is altered. Figure 10 displays the effects of modifying one bit in the original picture and how it affected the encryption result. Figure 10a shows the result of encrypting the Lena image without modifying, and Fig. 10b shows the result of encrypting the image after changing the most significant bit of pixel ${m}_{4\times 7}$.

By repeating the same test on other evaluated images, the average absolute value of the total difference was calculated as 23.199. The same criterion for the method presented in Ref.^15^ is equal to 9.221; for the method presented in Ref.^16^ it is equal to 0.12; and for the method presented in Ref.^17^ is equal to 2.788.

Sensitivity to plaintext is a necessary characteristic of a good encryption scheme [60]. A slight alteration to the plaintext will result in a significant change to the ciphertext. NPCR (Number of Point Changes Rate) and UACI (Unified Average Changing Intensity)^17^ are typically used to examine the differences between two images. Its definition is provided by the following Equations:

13 $$NPCR=100\times \frac{\sum_{i=0}^{M-1}\sum_{j=0}^{N-1}D(i,j)}{M\times N},$$

14 $$UACI=100\times \frac{\sum_{i=0}^{M-1}\sum_{j=0}^{N-1}\frac{|E\left(i,j\right)-{E}^{\prime}\left(i,j\right)|}{255}}{M\times N}.$$

The pixel gray values of two encrypted pictures at coordinates (i, j) are represented by $E(i, j)$ and $E^{\prime}(i, j)$, respectively; M and N stand for the image’s height and width, respectively. The definition of $D(i, j)$ is as follows: $D(i, j) = 1$ if $E(i, j) = E^{\prime}(i, j)$; $D(i, j) = 0$ otherwise. The expected results for these metrics are NPCR = 99.6094% and UACI = 33.4635%^17^. The disparity between the ciphertexts increases with bigger values of UACI and NPCR. In this experiment, values of the pixels in the dataset images were changed randomly and then, these criteria were calculated. This operation was repeated 200 times. Table 4 shows the average values obtained for UACI and NPCR in this experiment. The results reported in this Table demonstrate the ability of the proposed encryption algorithm against differential attacks.

**Table 4:** Average values of UACI and NPCR.

	Proposed	Ref.^15^	Ref.^16^	Ref.^17^
Average UACI	33.4629	33.4627	33.4400	33.4615
Average NPCR	99.6090	99.6090	99.6050	99.6082

### Key sensitivity

The smallest amount of change in the key that can both change the encryption output and not be able to recover the encrypted information is called the key sensitivity. Evaluation of the key sensitivity in an encryption method can be done in two ways:

- By making a very small change in the critical value, the resulting encrypted image must be completely different from the previous output.
- The original image must not be obtained by decrypting an image with a very slightly different from the valid key.

**Figure 11:**
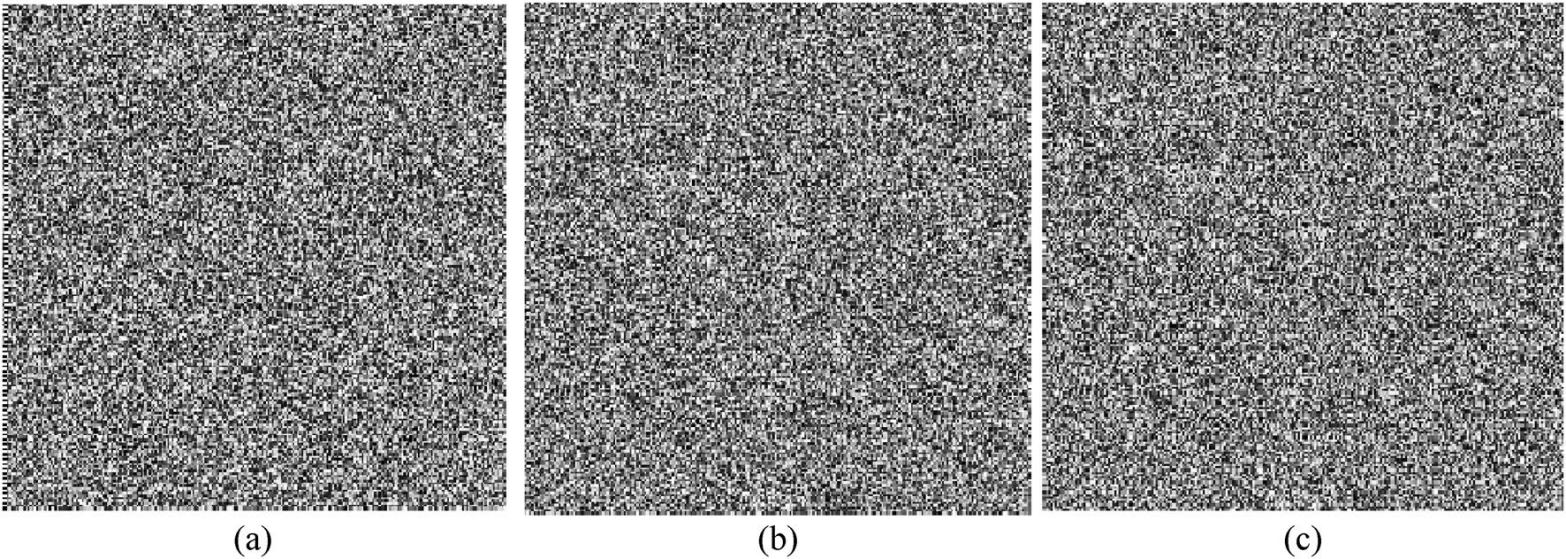
Key sensitivity evaluation: (**a**) encrypted image with key p = 0.5. (**b**) the same image encrypted with key $\mathrm{p}=0.5-1\times {10}^{-25}$. (**c**) Decrypted image with the wrong key $\upvarepsilon =0.300000000000000001$.

In Fig. 11a; the resulting encrypted image with the key *p* = 0.5 is displayed. If we encrypt the same image with a change of ${10}^{-25}$ in key *p*; image (Fig. 11b) is obtained, which is very different from image (Fig. 11a). This test shows that the *p* key has a sensitivity of ${10}^{-25}$ in the confusion process. On the other hand, if we want to restore the image (Fig. 11a) with a slight change of ${10}^{-18}$ for the *ε* key, we will get the image (Fig. 11c), based on which, the *ε* key has a sensitivity of ${10}^{-18}$ for the decryption process. Thus, the sensitivity of the key in the proposed method will be at least equal to ${10}^{-25}\times {10}^{-18}={10}^{-43}$. This high sensitivity in the proposed method can be seen as the result of using the spatiotemporal chaos model.

The results show that the proposed algorithm has high key sensitivity and can provide a high level of resistance against Brute–Force attacks.

### Entropy test

The entropy of information is considered one of the key criteria in measuring the randomness of information. Information entropy H(m) is calculated using the following equation^25^:

15 $$H\left(m\right)= -\sum_{i=1}^{{2}^{n}-1}p\left({m}_{i}\right){\mathrm{log}}_{2}\frac{1}{p\left({m}_{i}\right)},$$

where *m* represents the message and $p\left({m}_{i}\right)$ represents the probability of occurrence of sign ${m}_{i}$ in message *m*. In a message that is described by 8 bits (such as image pixel information that has a value between 0 and 2^8^), an ideal random state will have an entropy value of 8. Thus, an ideal random data for this case will have an entropy value close to 8. Therefore, the closer the resulting value is to 8, the more ideal the data will have a random structure.

The encrypted images produced by the proposed approach are closer to the ideal random state, as shown by the findings shown in Table 5, where it is closer to 8. These findings demonstrate that decrypting data using the suggested approach is more challenging than decrypting data using other examined methods. To attain high ciphertext information entropy, the suggested encryption technique makes use of two essential elements:

- *Two-way chaos maps* The complicated dynamic behavior of these maps results in wildly unpredictable sequences. We introduce a large amount of unpredictability into the encrypted image’s pixel values by encrypting these sequences.
- *RCA* RCAs have good diffusion qualities and can effectively jumble the values of all the pixels in the image. By further upsetting any possible statistical patterns in the original image, this diffusion process increases the unpredictability of the encrypted data.

**Table 5:** Comparison of the results of entropy analysis of encrypted images.

	Proposed	Ref.^15^	Ref.^16^	Ref.^17^
Lena	7.9974	7.9962	7.9883	7.9924
Camera man	7.9972	7.9967	7.9401	7.9556
pepper	7.9970	7.9873	7.9867	7.9933
Dataset average	7.9969	7.9934	7.9717	7.9804

The results in Table 5 show how these two factors interact to guarantee that the encrypted image has a high information entropy value. When compared to other techniques studied, encryption with entropy values closer to 8 is considered to be more random and secure.

### Time complexity and processing time analysis

This section examines the time complexity and processing time of the suggested image encryption algorithm in order to provide a thorough assessment of its effectiveness. Comprehending these facets is crucial for real-world scenarios where encryption speed is an essential element.

#### Time complexity analysis

The term “time complexity” describes how long an algorithm takes to run while the size of the input rises. The image size (n) is the input size in this case. To ascertain the total time complexity, we examine the key operations at each stage of the encryption procedure.

- *Step 1—converting image matrix to vector* Iterating over a $n\times n$ matrix to create a vector has a complexity of $O\left({n}^{2}\right)$, where n is the image size ($n\times n$ pixels).
- *Step 2—initial diffusion* This step involves bitwise XOR operations between elements, resulting in a complexity of $O(n)$.
- *Step 3—chaotic sequence generation and sorting* Generating a chaotic sequence might involve iterative calculations. The specific complexity depends on the chosen chaotic map which equals $O(n)$. Sorting the sequence using efficient algorithms like quicksort or merge sort has a complexity of $O(n\times log n)$.
- *Step 4—spatiotemporal chaotic matrix generation and processing* Generating the spatiotemporal chaotic matrix involves calculations based on the chaotic sequence elements. This operation has a complexity of O(n) (dependent on the previously generated sequence with $O(n)$ complexity). Extracting sub-sequences and performing modular operations contribute an additional complexity of $O(k)$, where k is the number of elements extracted.
- *Step 5—second diffusion* Similar to step 2, this step involves bitwise XOR operations, leading to a complexity of $O(n)$.
- *Step 6—RCA application* Iterating over each pixel and converting it to binary can be done in constant time ($O(1)$) for a fixed number of bits (4 in the proposed method). Applying RCA rules on each bit also involves constant time operations. Let’s denote the number of RCA iterations as ‘r’. The complexity for RCA on a single pixel becomes $O(r)$.

The most dominant term determines the total time complexity. The encryption algorithm’s overall complexity can be calculated as $O(n\times {\mathrm{log}} n)$, as the most time-consuming steps are sorting ($O(n\times {\mathrm{log}} n)$) and the production of a chaotic sequence ($O(n)$).

#### Processing time analysis

Processing time is the real wall-clock time required for encryption and decryption on a particular machine, whereas time complexity offers a theoretical knowledge of execution time based on input size. We evaluated the encryption durations for image of size $256\times 256$ on the computer running 64-bit Microsoft Windows 11 on an Intel Core i7 13700 CPU with 2.40 GHz processing power and 8 GB of memory in order to assess the processing time of our method. For comparison, these findings are shown in Table 6 with the processing times of methods^15–17^.

**Table 6:** Comparison of the results of processing time.

	Proposed	Ref^15^	Ref^16^	Ref^17^
Encryption time (s)	0.5121	2.4600	0.1345	0.9473
Machine configuration	MATLAB 2019a, CPU 2.4 GHz, 8 GB memory	MATLAB R2017a, CPU 2.8 GHz, 8 GB memory	MATLAB 2019a, CPU 2.6 GHz, 8 GB memory	–

As shown in Table 6, the encryption time of the proposed method is under 1 s and shorter than methods presented in Refs.^15,17^. We can ascertain that the suggested solution is comparable with current methods in terms of execution speed by comparing the processing times.

## Conclusion

The spatiotemporal chaos model is used in the suggested technique to ascertain the permutation pattern of image pixels. The pixel values of the muddled picture are also changed by the reversible cellular automata. The proposed spatiotemporal chaos model can efficiently solve problems such as small key space, low key sensitivity, and the chaotic sequence’s predictability. On the other hand, reversible cellular automata work effectively in facing the problem of high correlation of pixels. This combination led to the formation of an image encryption algorithm, whose efficiency was evaluated using various experiments. The intensity distribution of the pixels in the encrypted picture is sufficiently uniform and resembles a random distribution, according to the findings of the suggested algorithm’s histogram analysis. The suggested technique is, hence, well protected against statistical assaults. Since the suggested encryption technique may be applied to a variety of data types, its effectiveness in encrypting various forms of data (such as audio, text, and video) can be assessed in the next studies.

## Data Availability

All data generated or analyzed during this study are included in this published article.
